# Mimicking Facial Expressions Facilitates Working Memory for Stimuli in Emotion-Congruent Colours

**DOI:** 10.3390/vision8010004

**Published:** 2024-01-30

**Authors:** Thaatsha Sivananthan, Steven B. Most, Kim M. Curby

**Affiliations:** 1 School of Psychological Sciences, Macquarie University, Sydney, NSW 2109, Australia; 2 Macquarie University Performance & Expertise Research Centre, Macquarie University, Sydney, NSW 2109, Australia; 3 School of Psychology, University of New South Wales, Sydney, NSW 2052, Australia

**Keywords:** emotion, colour, emotional faces, colour–emotion associations, visual working memory

## Abstract

It is one thing for everyday phrases like “seeing red” to link some emotions with certain colours (e.g., anger with red), but can such links measurably bias information processing? We investigated whether emotional face information (angry/happy/neutral) held in visual working memory (VWM) enhances memory for shapes presented in a conceptually consistent colour (red or green) (Experiment 1). Although emotional information held in VWM appeared not to bias memory for coloured shapes in Experiment 1, exploratory analyses suggested that participants who physically mimicked the face stimuli were better at remembering congruently coloured shapes. Experiment 2 confirmed this finding by asking participants to hold the faces in mind while either mimicking or labelling the emotional expressions of face stimuli. Once again, those who mimicked the expressions were better at remembering shapes with emotion-congruent colours, whereas those who simply labelled them were not. Thus, emotion–colour associations appear powerful enough to guide attention, but—consistent with proposed impacts of “embodied emotion” on cognition—such effects emerged when emotion processing was facilitated through facial mimicry.

## 1. Introduction

Metaphors in everyday language frequently imbue otherwise neutral stimuli and concepts with emotional meaning. Colour is a prominent example: for example, you may be so angry that you “see red”. Green, for its part, has sometimes been linked with positive emotional experiences, as in naturalist John Muir’s statement that “nature in her green, tranquil woods heals and soothes all afflictions.” Research has suggested that links between colour and emotion extend beyond intuition and folk psychology, measurably impacting behaviour both in and out of the lab (e.g., [1,2], also see the work presented in [3] for cultural differences in colour–emotion associations).

Effects of colour on the processing of face stimuli have been consistent with such links between colour and emotion (e.g., [1,4]). For example, in a speeded emotion categorisation task, angry faces were more quickly categorised when they were primed with the colour red than green or grey before the onset of the face stimulus [5]. Redness has also been found to increase the perceived dominance and aggression of men’s faces [6], consistent with findings that wearing red is associated with a higher probability of winning across a range of sports [2]. In contrast, green backgrounds, compared to grey backgrounds, enhanced sensitivity to happy face information within neutral–happy morphed facial expressions [7], and produced more positive valence judgements about emotionally ambiguous neutral and surprised, facial expressions [8]. In short, studies have not only found consistent links between colours and emotions, but also have found that colours can facilitate the processing of emotional information when the colour and emotion are semantically congruent with each other (e.g., also see [9,10,11]).

It is not entirely clear whether such effects are bidirectional [1,12], with emotions influencing how people attend to and process colour stimuli that themselves have no evident emotional content. This study addressed this possibility by building on evidence that attention gravitates towards items or features held in visual working memory (VWM; [13,14,15,16]). For example, holding a face in VWM was found to bias selective attention for that image over another competing novel face image, even when attention to the faces was task-irrelevant [14].

Given this proposed functional link between items held in mind and the guidance of attention, this study investigated whether holding an emotional face in VWM leads to better memory for shapes in an emotion-congruent colour than for shapes in an emotion-incongruent colour. If colour–emotion associations are robust, it may be that holding an emotional stimulus in mind can lead to the preferential processing of, and consequently better memory for, stimuli in emotion-congruent colours. Specifically, we hypothesise that angry faces held in VWM will guide attention to, and memory for, red shapes compared to green shapes. Additionally, we hypothesise that happy faces held in VWM will guide attention to, and memory for green shapes over red shapes. The accurate retention of the facial expression by participants serves as the critical manipulation in our study. Accurate recognition of the faces is essential to assess the potential influence of emotionality held in VWM on the memory for shapes.

## 2. Experiment 1

### 2.1. Method

#### 2.1.1. Transparency and Openness

We report how we determined our sample size, all data exclusions (if any), all manipulations, and all measures in the study in alignment with JARS [17]. All data are available via Open Science Framework (OSF) data repository at https://bit.ly/3jiGVZp (accessed on 21 February 2021). Data were analysed using JASP [18]. This study’s design and analyses were not pre-registered.

#### 2.1.2. Participants

Forty-one undergraduate psychology students (16 females; *M_age_* = 20.32, *SD_age_* = 3.99) at Macquarie University participated for course credit. As we had no means of estimating what effect size to expect for this first experiment, we used a sample size that was typical in our lab. Participants reported normal or corrected to normal eyesight and no colour-blindness. The experiment was conducted in accordance with a protocol approved by the Macquarie University Human Research Ethics Committee.

#### 2.1.3. Materials and Stimuli

Eighteen Caucasian male identities with neutral (closed mouth), angry (open-mouth), and happy (open-mouth) facial expressions were used as stimuli from the NimStim face database [19]. Nine complex novel shapes used in previous research were also used as stimuli [20,21]. Each shape was coloured using the HSB colour model (red {0, 87, 72}, green {120, 87, 72}, and grey {0, 0, 72}) and presented on a black {0, 0, 0} background. Default hue values were used for red (0) and green (120), the values for the saturation (87) and brightness (72) were obtained from Elliot et al. (2007) [22]. The study was conducted on Intel Xeon processors presented on 24-inch BenQ XL2420T monitors with a 60 Hz refresh rate. All monitors were routinely colour-calibrated using Datacolor’s Spyder-3 Elite. The study was performed using E-Prime version 2.0 [23].

#### 2.1.4. Design and Procedure

**Figure 1:**
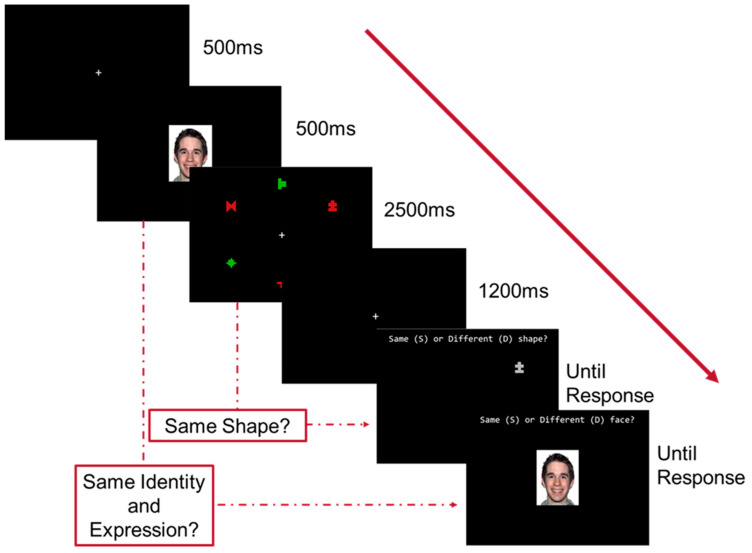
Progression of each trial.

Participants were seated approximately 60 cm from a monitor in a dimly lit room. Participants were informed that they would be tested on their memory for two separate tasks that were interweaved together; namely, a face and a shape memory task (see Figure 1).

The study started with 12 practice trials, followed by 6 blocks of 54 trials. The experimental design was a 3 (emotional expression; angry, happy, neutral) by 2 (shape colour; red, green), within subjects’ design. Each trial started with a centralised fixation cross followed by a face stimulus (9 cm × 7 cm) with either an angry, happy, or neutral facial expression presented for 500 ms. Participants were to hold the identity and expression of the face in memory. Following this, an array of six shapes (each 1.6 cm × 1.6 cm; three red and three green) equally spaced (subtending approximately 7.6 cm) around a centralised fixation cross were presented for 2500 ms; these complex shapes required greater encoding time than simple shapes [24]. Participants were told to ignore the colour of the stimuli as only their shape was task relevant. Following a blank screen for 1200 ms, a grey shape [25] was presented in one of six array positions. Participants performed a keyboard response reporting whether the shape of the grey stimulus was the same (‘S’) or different (‘D’) as the shape of the coloured stimulus that had been presented in that location during the shape study array. Once a response was registered, a face was then presented until a response and participants responded as to whether this face was the same (in identity and expression; ‘S’) or different (‘D’) as the face that was presented before the shape array (see Figure 1). For each participant, there is an equal number of same and different trials for both the shape and face tasks. Accuracy was recorded for the face and shape trials. Response time (RT) data was recorded for the shape trials and RT cut-offs were established to reduce the likelihood of spurious responses.

At the end of the study, participants were debriefed and asked a series of questions about strategies they might have used to remember the shapes and faces, including whether they had used a strategy to systematically focus on shapes of one colour over the other. Further, participants were assessed for whether they had guessed the purpose of the study. The following questions were administered 1. “Did you use any strategies to help you remember the shapes?”, 2. “Did you use any strategies to help you remember the faces?”, if participants responded ‘yes’ to either question a follow-up statement was asked “describe the strategy you used”, 3. “Do you know what the purpose of this experiment might be?”, if participants responded ‘yes’ a follow-up question was asked, “what do you think we might be testing?”. Participants who reported relying on a systematic preference for one colour over another or who guessed the correct purpose of the study were to be removed prior to statistical analyses, but no participants fell into either of these categories.

#### 2.1.5. Statistical Analysis

Participants’ raw data for the face memory accuracy and shape memory accuracy were converted to a proportion of accuracy for each of the six conditions (3 emotions by 2 colours). Participants were removed (*n* = 10) if they did not meet one or both accuracy cut-offs for the face (80% accuracy; 9 participants) or shape (55% accuracy; 3 participants) memory tasks (2 participants failed to meet threshold on both tasks), leaving 31 participants (11 females; *M_age_* = 20.61, *SD_age_* = 4.40). Accuracy and RT cut-offs were determined based on a pilot study. The pilot study revealed participants (*n* = 5) had an average face accuracy of 90.80% and an average shape accuracy of 67.16%. Thus, we determined a cut-off of 80% for the easier face accuracy task and 55% for the harder shape accuracy task. A high accuracy cut-off was necessary for the face task, as this ensured participants were engaged in the critical manipulation. The accuracy cut-off of the shape task was lower, reflecting the greater difficulty of this task. Trials for the shape task with an RT below 200 ms (89 trials) and two standard deviations above the average RT (5500 ms; 304 trials) were removed (2.96% of all data). Participants were required to hold the correct emotional face in memory to investigate the effect of emotional faces on shape memory prioritisation. Therefore, the shape task analyses consisted of shape response trials for which participants responded correctly to the face task in that trial. Multiple comparisons were corrected through a Bonferroni correction, and a Greenhouse–Geisser correction was applied where sphericity was violated. Consistent with Lakens (2013), 90% confidence intervals (CIs) were reported for ANOVAs and 95% CI were reported for *t*-tests [26].

### 2.2. Results

**Figure 2:**
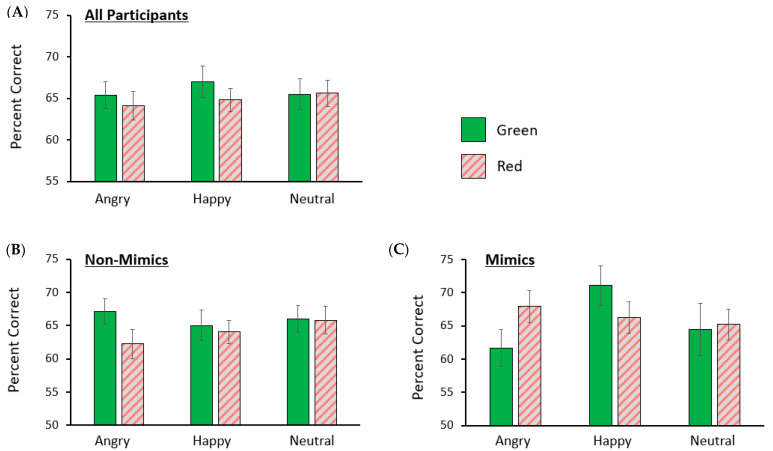
Memory accuracy for coloured shapes in Experiment 1 following the presentation of an expressive face (**A**) collapsed across self-selected memory strategy, among (**B**) non-mimickers (*n* = 21), and (**C**) mimickers (*n* = 10). Error bars represent the standard error of the mean.

A one-way repeated measures analysis of variance (ANOVA) was conducted to determine whether the emotionality of the facial expressions systematically influenced accuracy for face memory. There were no differences in working memory for faces across emotional expressions *F*(1.67, 50.11) = 0.59, *p* = 0.528, ƞ_p_^2^ = 0.02, 90% CI [0.00, 0.10]. To determine whether exposure to emotional faces biased working memory for shapes of certain colours, we conducted a three (emotion) by two (colour) within subjects’ ANOVA on response data from the shape memory task. There were no significant main effects of emotional expression, *F*(2, 60) = 0.46, *p* = 0.636, ƞ_p_^2^ = 0.02, 90% CI [0.00, 0.07], or colour, *F*(1, 30) = 1.12, *p* = 0.298, ƞ_p_^2^ = 0.04, 90% CI [0.00, 0.19], on memory for shapes. Further, the interaction between emotional expression and colour was not significant, *F*(2, 60) = 0.51, *p* = 0.606, ƞ_p_^2^ = 0.02, 90% CI [0.00, 0.08] (see Figure 2).

#### Post hoc Analysis

Participants’ post-task feedback revealed that they tended to adopt one of two distinct strategies for remembering the face stimuli. Ten participants (5 females; *M_age_* = 20.20, *SD_age_* = 3.99) reported that they had spontaneously mimicked the facial expression of the face presented at the beginning of each trial. Of the remaining (non-mimicking) 21 participants (6 females; *M_age_* = 20.81, *SD_age_* = 4.66), 17 reported using a labelling strategy for remembering the faces (e.g., “grumpy man”), with 4 reporting using no particular strategy. These participants were grouped together into the non-mimicking group.

We ran a 3 (emotional expression) by 2 (mimicking vs. non-mimicking) post hoc ANOVA on the data to determine whether the self-reported strategy moderated working memory for faces. The assumptions for the ANOVA were met. No significant main effect of emotional expression, *F*(1.65, 47.84) = 0.89, *p* = 0.400, ƞ_p_^2^ = 0.03, 90% CI [0.00, 0.12] or interaction between emotional expression and strategy, *F*(1.65, 47.84) = 2.07, *p* = 0.145, ƞ_p_^2^ = 0.07, 90% CI [0.00, 0.18], emerged. We also ran a 3 (emotional expression) by 2 (colour) by 2 (mimicking vs. non-mimicking) post hoc ANOVA on response data from the shape memory task. No significant main effects of emotional expression, *F*(2, 58) = 1.06, *p* = 0.354, ƞ_p_^2^ = 0.04, 90% CI [0.00, 0.12], colour, *F*(1, 29) = 0.32, *p* = 0.577, ƞ_p_^2^ = 0.01, 90% CI [0.00, 0.13], or strategy, *F*(1, 29) = 0.15, *p* = 0.703, ƞ_p_^2^ = 0.01, 90% CI [0.00, 0.11], emerged. Importantly, there was a significant interaction between emotional expression, colour, and strategy, *F*(2, 58) = 5.59, *p* = 0.006, ƞ_p_^2^ = 0.16, 90% CI [0.03, 0.28] (see Figure 2). No other interactions emerged, *p*s ≥ 0.121. To further probe the interaction between colour, emotional expression, and strategy, we conducted a 3 (emotional expression) by 2 (colour) within subjects’ ANOVA for the mimicking condition, followed by paired sample *t*-tests. A significant interaction between colour and emotional expression was found, *F*(2, 18) = 5.04, *p* = 0.018, ƞ_p_^2^ = 0.36, 90% CI [0.04, 0.53]. Paired sample *t*-tests revealed significantly higher shape accuracy for red (*M* = 0.68, *SD* = 0.08) than green (*M* = 0.62, *SD* = 0.09) shapes when mimicking angry facial expressions, *t*(9) = 2.67, *p* = 0.026, *d* = 0.84, 95% CI [0.10, 1.56], but no difference between red (*M* = 0.66, *SD* = 0.08) and green (*M* = 0.71, *SD* = 0.10) shapes when participants mimicked happy facial expressions, *t*(9) = 1.72, *p* = 0.120, *d* = 0.54, 95% CI [−0.14, 1.20]. However, working memory for shapes presented in green when participants mimicked happy expressions (*M* = 0.71, *SD* = 0.10) was better than when they mimicked angry expressions (*M* = 0.62, *SD* = 0.09), *t*(9) = 3.40, *p* = 0.008, *d* = 1.08, 95% CI [0.27, 1.85]. There was no significant difference in working memory for red shapes when participants mimicked angry expressions (*M* = 0.68, *SD* = 0.08) than when mimicking happy expressions (*M* = 0.66, *SD* = 0.08), *t*(9) = 0.82, *p* = 0.436, *d* = 0.26, 95% CI [−0.38, 0.88]. Intriguingly, a 3 (emotional expression) by 2 (colour) within-subjects’ ANOVA for accuracy among the non-mimickers did not find a significant interaction between colour and emotion, *F*(2, 40) = 1.75, *p* = 0.187, ƞ_p_^2^ = 0.08, 90% CI [0.00, 0.21].

### 2.3. Discussion

Experiment 1 investigated whether holding an emotional face in mind biases working memory in favour of emotion-congruent coloured shapes. Initial analyses did not support our hypothesis; merely holding emotional faces in VWM failed to produce a memory advantage for emotion-congruent coloured novel shapes (angry–red, happy–green). A review of participant feedback information regarding the strategies used to remember the faces and shapes revealed two predominant strategies. Some participants mimicked the facial expression of the face to be remembered, whereas other participants used a labelling strategy (or reported no strategy at all).

Intriguingly, an exploratory post hoc analysis split by participant strategy showed clear effects of emotion guiding memory for emotion-congruent coloured shapes in the mimickers, but not the non-mimickers. The small number of participants who mimicked the angry faces showed better memory for red than green shapes. In addition, mimickers showed better memory for green shapes when a happy, compared to an angry, face was held in VWM. The non-mimicking group failed to show either pattern.

The emergence of this apparent impact of facial mimicry in our exploratory analyses engendered a reconsideration and reframing of our initial research question. Indeed, questions about whether contents of working memory guide attentional selection are, as of this writing, a matter of some debate [27,28], so it may be particularly difficult to observe indicators that contents of working memory bias attention in favour of stimuli related only through associative links. Instead, the finding that mimicking of facial expressions was associated with better working memory for shapes that were colour-congruent with the expressed emotions is consistent with notions of “embodied emotion”, whereby the processing of emotional information—such as that from faces—is facilitated when people re-enact the physical manifestation of that emotion [29]; also see the work presented in [30,31]. Experiment 2 followed up on the unexpected findings from Experiment 1, testing whether the impact of facial mimicry on working memory for emotion-congruent colours survived a confirmatory approach.

## 3. Experiment 2

In Experiment 2, we sought to replicate and confirm Experiment 1’s serendipitous finding that mimicking of emotional expressions held in mind biases working memory in favour of shapes that appear in emotion-congruent colours. Mimicking has been shown to facilitate the recognition of emotion in other’s faces, and people have been reported to spontaneously mimic the facial expressions of others (e.g., [32,33]), especially people high in emotional empathy [34]. Thus, one question is whether the effects in the mimicking group reflected characteristics of participants within this self-selected group, for example greater empathy [35] or cultural norms [36], or whether they were a result of the mimicking strategy itself. To mitigate potential confounding factors, such as the degree of empathy influencing participants’ natural inclination to mimic, we experimentally manipulated the strategy participants adopted, instructing them to either mimic or label the facial expressions they were holding in mind. We selected the labelling strategy as a suitable control condition in Experiment 2, as it entailed greater engagement with the emotional stimuli than merely viewing the images would. Further, labelling was the most commonly reported strategy in Experiment 1 (facilitating comparison between the two experiments), and adherence to this strategy could be monitored by the experimenter in a manner similar to the mimicking condition. All other key aspects of the experiment remained unchanged from Experiment 1. To recap, we expect to replicate the findings of Experiment 1 among participants who have been allocated to mimic facial expressions. Specifically, we hypothesise that participants allocated to the mimicking condition, as opposed to the labelling condition, will show greater memory prioritisation for shapes presented in emotion-congruent colours (angry–red; happy–green) compared to those presented in emotion-incongruent colours.

### 3.1. Method

#### 3.1.1. Participants

Recruitment criteria and reimbursement remained the same as in Experiment 1, with the addition of an Ishihara colour vision test administered at the end of the experiment to ensure that participants could discriminate red from green [37]. A power analysis using G*Power determined that a minimum sample size of 52 participants was required to achieve a power level of 0.85 with an effect size of 0.436 (the effect size in Experiment 1). A larger sample size of 63 participants (52 females; *M_age_* = 20.68, *SD_age_* = 3.21) was recruited, with participants randomly assigned to either a mimicking (*n* = 32) or labelling (*n* = 31) condition. The larger sample size was in anticipation of the possible removal of participants who failed to meet accuracy thresholds or colour vision test requirements.

#### 3.1.2. Design and Procedure

This study was a 3 (emotional expression) by 2 (colour) by 2 (strategy) mixed design, using the same materials, stimuli, and trial structure as in Experiment 1 (see Figure 1). Unlike Experiment 1, participants were randomly allocated to a mimicking or labelling strategy condition. To monitor adherence to the assigned strategy (and to address self-reported lack of adherence in a pilot study), participants were tested individually with the experimenter in the room.

In the mimicking condition, upon exposure to the initial face on each trial, participants were instructed to facially model the emotion signalled by its expression (angry, happy, or neutral). Participants were instructed to use their natural expression to express the emotion, i.e., how they would normally express happiness or anger on their face. Participants were to hold this facial expression until the end of the trial when they were tested on their memory for the face (i.e., to determine whether the identity and expression of the test face were the same as those of the initial target face). In contrast, participants in the labelling condition were asked to repeatedly articulate aloud the emotion expressed on the first face image (angry, happy, or neutral) until the end of the trial, at which point they were tested on their memory for the identity and expression in the same manner as were those in the mimicking condition.

### 3.2. Results

Of the 63 participants tested, 3 participants were excluded from further analysis for not meeting either one or both accuracy thresholds for the face task (80%; 2 participants) or shape task (55%; 2 participants; 1 participant failed to meet threshold on both tasks). These cut-offs were the same as those in Experiment 1 (and informed by a pilot study run prior to Experiment 1; see footnote 1). Analysis was conducted after the removal of shape trials with an RT under 200 ms and greater than two standard deviations above the mean (3000 ms; 696 trials). This resulted in the removal of 3.43% of trials.

After exclusions, there were 32 participants in the mimicking condition (25 female, *M_age_* = 20.22, *SD_age_* = 1.72) and 28 participants in the labelling condition (24 female, *M_age_* = 21.36, *SD_age_* = 4.39). For the face task, a 3 (emotional expression) by 2 (strategy) factorial mixed-design ANOVA was conducted on the face accuracy data to determine whether the strategy adopted influenced face accuracy. There was no main effect of strategy on face accuracy, *F*(1, 58) = 0.76, *p* = 0.387, ƞ_p_^2^ = 0.01, 90% CI [0.00, 0.10], or interaction between strategy and emotional expression, *F*(1.78, 103.45) = 0.75, *p* = 0.462, ƞ_p_^2^ = 0.01, 90% CI [0.00, 0.06]. However, there was a main effect of emotional expression, *F*(1.78, 103.45) = 3.49, *p* = 0.039, ƞ_p_^2^ = 0.06, 90% CI [0.00, 0.13], with follow-up *t*-tests revealing better accuracy for neutral (*M* = 0.92, *SD* = 0.05) than angry (*M* = 0.91, *SD* = 0.05) faces, *t*(59) = 2.87, *p* = 0.006, *d* = 0.37, 95% CI [0.11, 0.63], but no significant difference between neutral (*M* = 0.92, *SD* = 0.05) and happy (*M* = 0.92, *SD* = 0.05) faces, *t*(59) = 0.42, *p* = 0.674, *d* = 0.06, 95% CI [−0.20, 0.31], and happy (*M* = 0.92, *SD* = 0.05) and angry (*M* = 0.91, *SD* = 0.05) faces, *t*(59) = 1.84, *p* = 0.071, *d* = 0.24, 95% CI [−0.02, 0.49].

**Figure 3:**
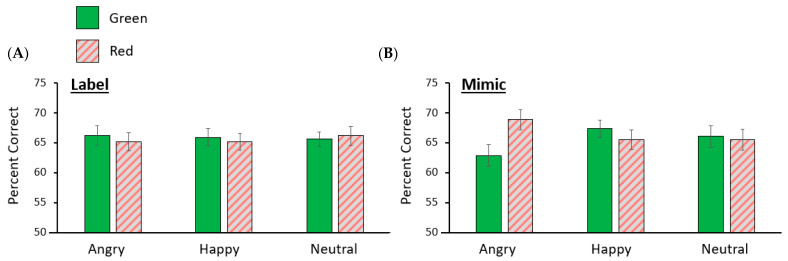
Mean memory accuracy for coloured shapes in Experiment 2 following the presentation of an expressive face among participants allocated to the (**A**) labelling, and (**B**) mimicking conditions. Error bars represent the standard error of the mean.

To determine whether the assigned strategy moderated the effects of facial expression on working memory for coloured shapes, a 3 (emotional expression) by 2 (colour) by 2 (strategy) mixed-design ANOVA was conducted on the data from those shape trials where participants correctly responded to the concurrent face task. The assigned strategy did not influence overall shape accuracy, *F*(1, 58) = 0.04, *p* = 0.84, ƞ_p_^2^ < 0.001, 90% CI [0.00, 0.04]. As in Experiment 1, there were no main effects of emotional expression, *F*(2, 116) = 0.03, *p* = 0.975, ƞ_p_^2^ < 0.001, 90% CI [0.00, 0.00], or colour *F*(1, 58) = 0.35, *p* = 0.554, ƞ_p_^2^ = 0.01, 90% CI [0.00, 0.08]. However, replicating the findings of Experiment 1, there was an interaction between emotional expression, colour, and strategy, *F*(2, 116) = 3.53, *p* = 0.032, ƞ_p_^2^ = 0.06, 90% CI [0.00, 0.13] (see Figure 3). No other interactions emerged, *p*s ≥ 0.107. To investigate the interaction between colour and emotional expression in the mimicking condition, we conducted a 3 (emotional expression) by 2 (colour) within subjects’ ANOVA, which revealed a significant interaction, *F*(2, 62) = 5.47, *p* = 0.007, ƞ_p_^2^ = 0.15, 90% CI [0.03, 0.27]. Paired sample *t*-tests revealed significantly higher shape accuracy for red shapes (*M* = 0.69, *SD* = 0.10) than green shapes (*M* = 0.63, *SD* = 0.10) when mimicking angry facial expressions, *t*(31) = 3.19, *p* = 0.003, *d* = 0.56, 95% CI [0.19, 0.93], but no difference in accuracy between red (*M* = 0.66, *SD* = 0.09) and green (*M* = 0.67, *SD* = 0.10) shapes when mimicking happy facial expressions, *t*(31) = 1.00, *p* = 0.327, *d* = 0.18, 95% CI [−0.18, 0.52]. However, memory for shapes presented in green was better when participants mimicked happy faces (*M* = 0.67, *SD* = 0.08) than when they mimicked angry faces (*M* = 0.63, *SD* = 0.10), *t*(31) = 2.96, *p* = 0.006, *d* = 0.52, 95% CI [0.15, 0.89]. There was no significant difference in working memory for red shapes when participants mimicked angry expressions (*M* = 0.69, *SD* = 0.10) than when they mimicked happy expressions (*M* = 0.66, *SD* = 0.09), *t*(31) = 1.84, *p* = 0.076, *d* = 0.32, 95% CI [−0.03, 0.68]. Notably, as expected, a separate 3 (emotional expression) by 2 (colour) within subjects’ ANOVA for the labelling condition did not find a significant interaction, *F*(2, 54) = 0.26, *p* = 0.771, ƞ_p_^2^ = 0.01, 90% CI [0.00, 0.06].

### 3.3. Discussion

Experiment 2 confirmed the serendipitous findings of Experiment 1. Red shapes were better remembered than green shapes when people mimicked an angry facial expression, a difference that was not present when they mimicked happy facial expressions. In addition, there was evidence that memory for green shapes was also impacted by mimicking the emotional expressions: greens shapes were better remembered when people mimicked a happy than an angry facial expression. No such effects were found in the labelling condition. As in Experiment 1, there was no two-way interaction between colour and expression suggesting that the act of holding an emotional face in VWM did not guide memory for coloured shapes independently of the strategy used to maintain the emotional information. Further, there was no main effect of strategy, suggesting that the strategy adopted did not have a more general effect on memory performance.

## 4. General Discussion

The goal of this study was to determine whether holding an emotional stimulus in mind biases working memory in favour of colours that are emotion-congruent (e.g., red/angry, green/happy). Specifically, we tested whether holding angry, happy, and neutral facial expressions in mind affected visual working memory (VWM) for red and green novel shapes, which have been previously, metaphorically linked with angry and happy emotions, respectively [1,7,10]. At first glance, the results of Experiment 1 appeared to suggest that holding an emotional face in mind did not facilitate VWM for task-irrelevant, emotion-congruent coloured stimuli. However, post hoc analyses revealed a facilitation of VWM performance for emotion-congruent coloured shapes among participants who mimicked the facial expressions they were holding in mind. Experiment 2 confirmed this pattern by explicitly instructing participants to either mimic or simply label the emotional expressions they were holding in mind, with the result that those who mimicked the facial expressions had better VWM for the shapes in emotion-congruent colours.

The moderating effects of mimicry on VWM for shapes in emotion-congruent colours are consistent with evidence that emotion processing can be facilitated when people assume postures and facial expressions that are congruent with the emotion that is being processed. For example, moving one’s facial muscles in a manner consistent with a natural facial expression has been found to impact a person’s emotional state [38], alter the processing and evaluation of emotional stimuli [29,39], enhance emotion categorization [40], facilitate facial emotion processing [39,40,41,42,43], and influence encoding and maintenance of stimuli that are themselves emotional in VWM [44]. Moreover, induced changes to participants’ emotional state biases the perception of ambiguous colours towards that consistent with their emotional state (e.g., anger–red; [4]). An open question from the present study is whether the greater bias towards emotion-congruent colours among those who mimicked the facial expressions might have stemmed from greater engagement with the emotion expressions (rendering them more salient in VWM and therefore more effective guides of attention; e.g., [45]) or emerged because such mimicking modulated participants’ emotional state in line with the facial feedback hypothesis (e.g., [31]). Research has found that emotion inductions can shift attentional resources towards stimuli congruent with the induced emotions (e.g., [46,47]). If the latter, then it is possible that mimicking facial expressions can bias working memory in favour of emotion-congruent colours even when people do not hold the faces themselves in working memory. This is a possibility that can be tested in future work.

It is also possible that the control strategy introduced in Experiment 2 had other, unexpected effects on colour memory. For example, this might be the case if the labelling task introduced more cognitive load than the mimicking task. However, there was no main effect of strategy in Experiment 2, suggesting that the absence of an effect of shape colour in the labelling condition is unlikely to be a result of this strategy being more distracting and/or more difficult for participants.

The emotionality of the faces also impacted participants’ memory for the faces, with lower performance in the angry than the neutral condition in the face memory task. One possible explanation for this pattern is that the angry faces drew more attention to the emotional information in the face and away from the identity-related information, which impaired performance in the memory task, where both types of information were required to accurately perform the task. The poorer VWM for faces with negative expressions is also consistent with previous findings from our lab [48], and are more generally consistent with reduced WM capacity for emotional than neutral stimuli [49]. Further experimentation is required to address this possibility. In addition, in both Experiments 1 and 2, the effect of facial expression on shape memory in the mimicking condition is driven by angry facial expressions. This effect may be attributed to a greater arousal associated with angry facial expressions compared to happy expressions [50]. Future research could explore the effects of other high arousal, negatively valenced emotions, such as fear. Future work may also usefully control for potential interactions between the sex of the participants and of the presented faces. In this study, all presented faces were male, so such interactions could not be explored.

The need for an experimenter to be physically present during testing to ensure that participants applied (and maintained) the strategy that they were assigned introduces the possibility of demand characteristics. Specifically, it is possible that the experimenter may have unintentionally influenced participant’s performance in the different conditions (e.g., [51]). However, given that the same pattern was found in Experiment 1, when no experimenter was present and participants were free to adopt their own strategy, this is unlikely to be the case.

In conclusion, merely holding an emotional face in VWM alone did not bias attention, and thus working memory, for emotion-congruent coloured shapes. However, mimicking emotional faces while holding them in mind did seem to do so. Although it may fall to future research to uncover the precise mechanisms by which such facial mimicry causes such biased processing, taken together, the current findings suggest that metaphorical links between colour and emotion are robust enough that attention to emotion can guide visual processing of colour, and they bolster existing evidence for the notion that “embodied” emotion processes play a measurable role in the processing of emotional and emotion-congruent information.

## Data Availability

The experiments reported in this article were not preregistered. The data is made available via Open Science Framework (OSF) data repository at https://bit.ly/3jiGVZp.

## References

[1] Fetterman A.K., Robinson M.D., Meier B.P. (2012). Anger as “seeing red”: Evidence for a perceptual association. Cogn. Emot..

[2] Hill R.A., Barton R.A. (2005). Red enhances human performance in contests. Nature.

[3] Jonauskaite D., Wicker J., Mohr C., Dael N., Havelka J., Papadatou-Pastou M., Zhang M., Oberfeld D. (2019). A machine learning approach to quantify the specificity of colour–emotion associations and their cultural differences. R. Soc. Open Sci..

[4] Fetterman A.K., Robinson M.D., Gordon R.D., Elliot A.J. (2011). Anger as seeing red: Perceptual sources of evidence. Soc. Psychol. Personal. Sci..

[5] Young S.G., Elliot A.J., Feltman R., Ambady N. (2013). Red enhances the processing of facial expressions of anger. Emotion.

[6] Stephen I.D., Oldham F.H., Perrett D.I., Barton R.A. (2012). Redness enhances perceived aggression, dominance and attractiveness in men’s faces. Evol. Psychol..

[7] Gil S., Le Bigot L. (2014). Seeing life through positive-tinted glasses: Color–meaning associations. PLoS ONE.

[8] Sivananthan T., de Lissa P., Curby K.M. (2021). Colour context effects on speeded valence categorization of facial expressions. Vis. Cogn..

[9] Kuhbandner C., Pekrun R. (2013). Joint effects of emotion and color on memory. Emotion.

[10] Mammarella N., Di Domenico A., Palumbo R., Fairfield B. (2016). When green is positive and red is negative: Aging and the influence of color on emotional memories. Psychol. Aging.

[11] Sutton T.M., Altarriba J. (2016). Color associations to emotion and emotion-laden words: A collection of norms for stimulus construction and selection. Behav. Res. Methods.

[12] Ikeda S. (2019). Influence of color on emotion recognition is not bidirectional: An investigation of the association between color and emotion using a stroop-like task. Psychol. Rep..

[13] Bahle B., Beck V.M., Hollingworth A. (2018). The architecture of interaction between visual working memory and visual attention. J. Exp. Psychol. Hum. Percept. Perform..

[14] Downing P.E. (2000). Interactions between visual working memory and selective attention. Psychol. Sci..

[15] Pashler H., Shiu L.-P. (1999). Do images involuntarily trigger search? A test of Pillsbury’s hypothesis. Psychon. Bull. Rev..

[16] van Moorselaar D., Theeuwes J., Olivers C.N. (2014). In competition for the attentional template: Can multiple items within visual working memory guide attention?. J. Exp. Psychol. Hum. Percept. Perform..

[17] Kazak A.E. (2018). Editorial: Journal article reporting standards. Am. Psychol..

[18] JASP Team (2020). JASP.

[19] Tottenham N., Tanaka J.W., Leon A.C., McCarry T., Nurse M., Hare T.A., Marcus D.J., Westerlund A., Casey B.J., Nelson C. (2009). The NimStim set of facial expressions: Judgments from untrained research participants. Psychiatry Res..

[20] Blacker K.J., Curby K.M. (2013). Enhanced visual short-term memory in action video game players. Atten. Percept. Psychophys..

[21] Fiser J., Aslin R.N. (2002). Statistical learning of higher-order temporal structure from visual shape sequences. J. Exp. Psychol. Learn. Mem. Cogn..

[22] Elliot A.J., Maier M.A., Moller A.C., Friedman R., Meinhardt J. (2007). Color and psychological functioning: The effect of red on performance attainment. J. Exp. Psychol. Gen..

[23] Schneider W., Eschman A., Zuccolotto A. (2002). E-Prime User’s Guide.

[24] Curby K.M., Gauthier I. (2007). A visual short-term memory advantage for faces. Psychon. Bull. Rev..

[25] Clarke T., Costall A. (2008). The emotional connotations of color: A qualitative investigation. Color Res. Appl..

[26] Lakens D. (2013). Calculating and reporting effect sizes to facilitate cumulative science: A practical primer for *t*-test and ANOVAs. Front. Psychol..

[27] Hollingworth A., Beck V.M. (2016). Memory-based attention capture when multiple items are maintained in visual working memory. J. Exp. Psychol. Hum. Percept. Perform..

[28] Woodman G.F., Luck S.J. (2007). Do the contents of visual working memory automatically influence attentional selection during visual search?. J. Exp. Psychol. Hum. Percept. Perform..

[29] Niedenthal P.M. (2007). Embodying emotion. Science.

[30] Chartrand T.L., Bargh J.A. (1999). The chameleon effect: The perception–behavior link and social interaction. J. Personal. Soc. Psychol..

[31] Strack F., Martin L.L., Stepper S. (1988). Inhibiting and facilitating conditions of the human smile: A nonobtrusive test of the facial feedback hypothesis. J. Personal. Soc. Psychol..

[32] Dimberg U., Thunberg M., Grunedal S. (2002). Facial reactions to emotional stimuli: Automatically controlled emotional responses. Cogn. Emot..

[33] Korb S., Grandjean D., Scherer K.R. (2010). Timing and voluntary suppression of facial mimicry to smiling faces in a Go/NoGo task—An EMG study. Biol. Psychol..

[34] Dimberg U., Thunberg M. (2012). Empathy, emotional contagion, and rapid facial reactions to angry and happy facial expressions. Psych. J..

[35] Dimberg U., Andréasson P., Thunberg M. (2011). Emotional empathy and facial reactions to facial expressions. J. Psychophysiol..

[36] Wood A., Rychlowska M., Korb S., Niedenthal P. (2016). Fashioning the face: Sensorimotor simulation contributes to facial expression recognition. Trends Cogn. Sci..

[37] Ishihara S. (1960). Tests for Colour-Blindness.

[38] Soussignan R. (2002). Duchenne smile, emotional experience, and autonomic reactivity: A test of the facial feedback hypothesis. Emotion.

[39] Wood A., Lupyan G., Sherrin S., Niedenthal P. (2016). Altering sensorimotor feedback disrupts visual discrimination of facial expressions. Psychon. Bull. Rev..

[40] Neal D.T., Chartrand T.L. (2011). Embodied emotion perception: Amplifying and dampening facial feedback modulates emotion perception accuracy. Soc. Psychol. Personal. Sci..

[41] Oberman L.M., Winkielman P., Ramachandran V.S. (2007). Face to face: Blocking facial mimicry can selectively impair recognition of emotional expressions. Soc. Neurosci..

[42] Ponari M., Conson M., D’Amico N.P., Grossi D., Trojano L. (2012). Mapping correspondence between facial mimicry and emotion recognition in healthy subjects. Emotion.

[43] Rychlowska M., Cañadas E., Wood A., Krumhuber E.G., Fischer A., Niedenthal P.M. (2014). Blocking mimicry makes true and false smiles look the same. PLoS ONE.

[44] Sessa P., Schiano Lomoriello A., Luria R. (2018). Neural measures of the causal role of observers’ facial mimicry on visual working memory for facial expressions. Soc. Cogn. Affect. Neurosci..

[45] Moores E., Laiti L., Chelazzi L. (2003). Associative knowledge controls deployment of visual selective attention. Nat. Neurosci..

[46] Becker M.W., Leinenger M. (2011). Attentional selection is biased toward mood congruent stimuli. Emotion.

[47] Cavanagh S.R., Urry H.L., Shin L.M. (2011). Mood-induced shifts in attentional bias to emotional information predict ill-and well-being. Emotion.

[48] Curby K.M., Smith S.D., Moerel D., Dyson A. (2019). The cost of facing fear: Visual working memory is impaired for faces expressing fear. Br. J. Psychol..

[49] Garrison K.E., Schmeichel B.J. (2019). Effects of emotional content on working memory capacity. Cogn. Emot..

[50] Vasara D., Surakka V. (2021). Haptic responses to angry and happy faces. Int. J. Hum.-Comput. Interact..

[51] Rosenthal R., Jacobson L. (1968). Pygmalion in the Classroom: Teacher Expectations and Student Intellectual Development.

